# Books and Magazines

**Published:** 1903-12

**Authors:** 


### Books and Magazines

THE MEDICAL NEWS VISITING LIST FOR 1904. An invaluable, pocket-sized, wallet-shaped book, containing memoranda and data important for every physician, and ruled blanks for recording every detail of practice.

It is in its eighteenth year of issue. *The Medical News Visiting List* embodies the results of long experience and study devoted to its development and perfection. It is up to its usual standard of excellence.

RADAEROTHERAPHY: LIQUID AIR AND THE X-RAY IN MEDICINE AND SURGERY. Reprints of papers by Dr. A. Campbell White, in *Medical Record, Journal American Medical Association, Interstate Medical Journal,* and *Medical Mirror.*

Dr. White has established a "Liquid Air and X-ray Institute" in New York for the use of physicians for the treatment of diseases of the skin, malignant and non-malignant, by these new therapeutic agents. Dr. White says that for "boils and carbuncles, liquid air is an absolute specific." This beautifully illustrated pamphlet is published by the Liquid Air and X-ray Institute, 537 Fifth avenue, New York, and a copy may be had free by mentioning this article.

